# Health, social, and civic society professionals' and volunteers' view on the health among young people in unstable housing situations in the south of Sweden: a qualitative interview study

**DOI:** 10.3389/fpubh.2025.1603322

**Published:** 2025-06-25

**Authors:** Elisabeth Mangrio, Maria Afzelius, My Lilja, John Stigmar, Annelie Björkhagen Turesson

**Affiliations:** ^1^Department of Care Science, Malmö University, Malmö, Sweden; ^2^Department of Social Work, Malmö University, Malmö, Sweden; ^3^Department of Criminology, Malmö University, Malmö, Sweden; ^4^Department of Social Work, Criminology and Public Health Sciences, University of Gävle, Gävle, Sweden

**Keywords:** civic society, health care, health, unstable housing, young people, qualitative study

## Abstract

**Background:**

Stable housing is important for overall health and well-being among young people, and poor-quality housing can trigger multiple diseases, including infections, injuries, chronic diseases, and psychological problems. However, many young people in Sweden struggle with an unstable housing situation. It is therefore important to illuminate how health, social, and civic society professionals and volunteers see the overall health situation for young people (16–27 years) in unstable housing situations.

**Method:**

Sixteen health, social, and civic society professionals and volunteers, from both municipal and governmental organizations and civic society, were interviewed during 2024 and were reached through convenience and purposive sampling. The data was analyzed with content analysis.

**Results:**

The results showed that the professionals and volunteers considered that there is a lack of basic necessities, such as food and clothes and often also a place to sleep, among young people in unstable housing situations. They also recounted that the bodies of those young people are weakened as a result of the unstable situation, with its stress and lack of proper care. There are, however, challenges with regard to accessing healthcare and the young people in question are often hesitant to seek care, and inclined to avoid it. Furthermore, the participants mentioned psychosocial challenges, due to loneliness and lack of close contacts with family and friends, and said that people struggling with homelessness tend to suffer from mental illness.

**Conclusion:**

Young people in unstable housing situations need support to stabilize them for the future, and in order to work toward a more stable housing situation for these people in the county of Scania, a holistic approach is necessary. This is crucial in view of their overall health, as well as their well-being and life satisfaction.

## 1 Introduction

Maintaining quality and adequate housing is critical in improving inhabitants' comfort, health, satisfaction, safety, and security. Poor-quality housing can trigger multiple diseases, including infections, injuries, chronic diseases, and psychological problems (1). For children, a stable home situation is a foundation for quality of life and health (2, 3). But a report from the National Board of Health and Welfare, published in 2024, states that around 27,000 people in Sweden are homeless, where homelessness is defined as one of the following situations: acute homelessness, staying in an institution, or having a very short-term home, e.g., staying with friends (4). The same report states that more knowledge around homelessness is needed, as well as more insight into the collaboration between the social services and the urban planning in municipalities (5).

In Malmö, where most of the interviews were conducted, 1,280 adults and 292 children were homeless in 2024, according to the Swedish City Missions' homelessness report (6). Some groups are excluded from the report, such as young people without a residence permit and those living in institutions. Also excluded are those categorized by the social services as structurally homeless, i.e., where the homelessness is not due to social problems. According to the report, most of both the homeless children and the homeless adults in Malmö live in a situation of acute homelessness, which means that they are referred to shelters or emergency accommodation, or live in tents and staircases (6).

Having a home is important for most people, and according to Jackson and Usher, “the word ‘home' holds profound existential meaning.” Home “represents a place of safety, shelter and comfort” as well as a place “for storage of essential items.” Furthermore, home “implies a sanctuary,” that is, somewhere one can relax and be oneself, and “should also be a place of belonging, providing a means of connection with family and friends” (7). Being deprived of a home is therefore a difficult situation.

There are different reasons why a child or young adult lives in an unstable housing situation; they could, for instance, have been forced to leave their apartment or had to leave a temporary housing situation at friends', or they could have lived with an illegal lease contract and therefore not been allowed to stay any longer (7, 8). Furthermore, some families could lose the contract of their apartment, and thereby their home, due to someone in the family being engaged in criminal activities. Then there are usually difficulties finding new housing due to the low number of available and affordable apartments in the different municipalities. There could also be challenges when newly arrived refugees receive their family members from the home country, which may make the apartment so crowded that they are not allowed to stay there (7). The most common reasons for homelessness in Malmö are substance abuse, mental illness, or not being able to compete in an overheated housing market (9). In this paper, we define homelessness as not having a first-hand contract on your own housing, meaning that the person does not have control over their own living situation.

Economic factors and infrastructure affect the mental health and well-being of individuals within a country. In the words of a WHO report, “mental health and well-being is influenced not only by individual attributes, but also by the social circumstances in which persons find themselves and the environment in which they live” (10). A review on social determinants of mental health, showed that there were associations between risks for worse mental health and individual factors, such as low income, not living with a partner, lack of social support, female gender, low level of education, low income, low socioeconomic status, unemployment, financial strain, and perceived discrimination (11). It also showed an association between worse mental health and area-level factors, such as neighborhood socioeconomic conditions, social capital, geographical distribution and built environment, neighborhood problems and ethnic composition (11). When preventing and targeting situations around homelessness, healthcare and social services should be aware of and understand risk factors on both individual and area levels. This is particularly important to consider in view of the increased risk for unstable and adverse housing conditions for young people with different mental health issues, as shown by previous research (10, 12).

The COVID-19 pandemic revealed some social challenges that were mostly seen among vulnerable groups in society in Sweden, where unemployment rose and there were increased challenges with unstable home situations for some people (13, 14). Migrants, a group in society that could be seen as vulnerable, had challenges with proper housing already before the COVID-19 pandemic (8). It was not the (frequently) crowded housing in itself that constituted the highest risk for mental ill-health but the instability of the housing, such as not having a proper contract and therefore having to move frequently (8, 15).

According to the United Nations' Convention on Human Rights, all people should have stable housing and a decent home. The EU has clarified what to consider when it comes to stable and decent housing, emphasizing that we should promote access to such housing, prevent and reduce homelessness, and make apartments accessible at lower costs (7).

Since health is related to basic needs, such as having stable housing, it is important to illuminate the perspective that health, social, and civic society professionals and volunteers working with young people have on how unstable housing conditions could affect the overall health of these young people, which is the aim of the current study. To the best of our knowledge, there are few studies on that particular perspective.

## 2 Method

The current research was conducted by a cross-disciplinary research group and a research platform called Child Health and Equity (16), with researchers within social work, criminology, and care science. Together we initiated research around unstable housing situations for young adults, and we planned and conducted interviews with health, social, and civic society professionals and volunteers. Qualitative research was the method chosen for the current study, since qualitative interviews are conducive to understanding subjective perspectives of phenomena, instead of broader, generalizable perspectives, and to investigating individual experiences (17). In the present study, the experiences that are investigated are those that health, social, and civic society professionals and volunteers have of how the overall health of young people could be affected by unstable housing situations.

### 2.1 Setting

The research focused on interviews with different health, social, and civic society professionals and volunteers that were part of the researchers' contact net within the county of Scania, in the south of Sweden. Various professionals and volunteers were contacted by the research team, and this could be considered a convenience and purposive sampling, since we reached out to professionals and volunteers that were within our contact net and that we expected to be knowledgeable about the topic under study. The strength of purposive sampling is the selection of informants that can provide rich information and from whom it is thus possible to learn very much about the issues that a study proposes to explore (18). Convenience sampling is non-probability sampling and frequently used in qualitative research. It means that the participants are chosen among those that are “available around a location” (21, p. 1), in the current study contacts around the county of Scania (19). The 19 informants that were interviewed in the study were volunteers and professionals within different health and social centers. Some were health professionals, such as nurses, but most were social workers working within different NGOs, including the Swedish church (a deacon), a shelter for homeless people, youth organizations with after-school activities, and the City Mission. Some professionals worked within mental health clinics, the municipalities of Malmö and Lund, and the social services. The inclusion criteria for the current study were that participants should have worked with and met young people in unstable housing situations. See the sample with the represented organizations in Table 1.

**Table 1:** Respondents and type of organization that they represented.

**Participant**	**Type of organization**
1	NGO
2	NGO
3	Region/municipal
4	Church/NGO
5	NGO
6	NGO
7	NGO
8	NGO
9	Municipal
10	NGO
11	NGO
12	NGO
13	Municipal
14	Municipal
15	Governmental organization
16	Municipal

### 2.2 Data collection

The professionals and volunteers that were within the contact net of the researchers, were emailed or phoned by one of the researchers. If reached, they were asked if they were willing to participate in an interview, and the time and place for the interview was booked. The first, second, third, and fifth author conducted the interviews, dividing them between the authors. They were carried out during 2024, and, in total, 16 interviews with 19 informants were performed. The interviews were semi-structured and the total interview time was between 45 and 60 min. An interview guide was developed within the research team, and it contained a broad range of questions in regard to the unstable housing situation for young adults (aged 16–27 years).

### 2.3 Analysis

The chosen method for analyzing the data in the study was an inductive content analysis approach, which means “analyzing data with little or no predetermined theory, structure, or framework and us[ing] the actual data itself to derive the structure of analysis” (17). In the current paper, only data related to the health situation, including mental health and psychosocial health, of the youth in unstable housing situations, was analyzed, and the rest of the data was saved for another analysis. The data that was related to the aim of this paper was coded by the first author, who read each transcript and made notes in the margins of words, theories, or short phrases that summed up what was being said in the text (17). This is usually known as open coding (17). Then the coded data was organized into groups with similar codes, and the first author looked for overlapping or similar categories (17). Subsequently, these groups were named and categorized into sub-categories. A main category was lifted from the five sub-categories. The analysis process followed the thematic content analysis by Burnard (17). See an example of the coding in Table 2.

**Table 2:** Analysis schedule with coding from meaning unit to code to sub-category.

**Meaning unit**	**Code**	**Sub-category**
Imagine that they have to sleep outside during the cold winter and lack warm covers. (Informant 6)	It is cold to sleep outside during the winter.	To lack basic necessities in life
Almost everyone feels insecure. They have some contact with each other but mostly unstable contacts. (Informant 2)	They feel insecure and have unstable contacts.	To be affected psychosocially

## 3 Results

Both volunteers and professionals working with young people in unstable housing situations, were interviewed. Some of them met children and teenagers, and for these persons the unstable housing situations mainly concerned whole families. Some met young adults (19–24 years), mostly migrants, who were in more or less homeless situations and some of whom also had addiction problems. The analysis of the results resulted in one main category and five sub-categories. The main category was *To live in an instable housing situation affects the overall health of a person*, and the subcategories were *To lack basic necessities in life, To be physically weak, To be affected psychosocially, To suffer from mental ill-health*, and *To struggle with healthcare access*.

### 3.1 To live in an unstable housing situation affects the overall health of a person

The main category arrived at in the analysis, covers how different aspects of an unstable housing situation affect the health of young people. In such a situation there is a lack of basic necessities, such as food and clothes as well as a place to sleep, and the body tends to get weak as a result of the instability of the living conditions. There also seem to be psychosocial challenges due to loneliness and lack of close contacts with family and friends. Moreover, persons in an unstable housing situation often suffer from mental ill-health, with stress symptoms, anxiety, or mental diagnoses. Furthermore, there appear to be challenges with regard to accessing healthcare and young homeless people often hesitate to seek care.

#### 3.1.1 To lack basic necessities in life

The professionals and volunteers described how the young people in unstable housing situations lacked basic things, such as clothes and food. For the young people who went to school, the food served in school was sometimes the only meal they got during the days and this could cause underweight or a lack of important nutritional components. The interviewees also mentioned that the homeless youth often lacked good shoes, which was something they really needed since they had to walk a lot. They also talked about how the lack of housing caused tiredness due to lack of sleep, and how it meant a lack of basic hygiene, which one person expressed as follows:

*One boy came to our place and had been out the whole night. He was dirty and lacked clean clothes and needed to shower and he also needed toothpaste and a toothbrush. Imagine lacking these kinds of basic necessities in life*. (Informant 2)

Other professionals and volunteers said that they, as a church or a homeless shelter, were able to distribute food and clothes, describing how many there were that needed this support to manage basic needs in life.

#### 3.1.2 To be physically weak

The professionals and volunteers talked about how the unstable housing situation affected the physical health of these young people and could manifest as underweight, stomach ulcers, headaches, stomach pain, fibromyalgia, and neuralgia. The pain in the body that many homeless young people seemed to experience, was, among other things, related to walking a lot and often carrying around their household in bags, the participants said. They were also bothered by sleeplessness, and whenever they tried to sleep, it was often difficult for them to fall asleep. One of the participants said this:

*They have difficulties to sleep, and wake up with bad dreams and pain in the body, and probably they don't understand that it's related to all the trauma they've been through in life*. (Informant 5)

The professionals and volunteers also narrated how the stress experienced by these young people affected them somatically, usually during periods when they were feeling worse. Furthermore, they described how their dental health was affected. They also mentioned sore feet, as well as the suffering sometimes resulting from being in conflicts and fights, due to exposure to violence. The injuries from violence could sometimes be evident when the professionals met with these young people.

#### 3.1.3 To be affected psychosocially

The professionals and volunteers talked about the young people suffering from being, and feeling, excluded from society, and how this caused barriers between people. One participant described one kind of barrier between people:

*Before, there were unseen barriers between these people and the rest of society, but now they are more evident. This is created by the “visitation zones” that are created by the government to be able to check or examine people when they consider them being at risk of committing criminal acts*. (Informant 1)

The professionals and volunteers also raised the fact that homeless young people often lacked close family contacts as well as close friends. They stated that especially fathers were often absent from the young people's lives, and that mothers were not always very present either, as they frequently had several jobs, which meant that the children at home had to take care of themselves and each other. Moreover, the professionals and volunteers mentioned that there were a lot of conflicts between these young people. Most of them were on social media, which risked increasing the feeling of loneliness. One participant said that although these young people often had lots of interactions, they lacked close social contacts and real friends. Furthermore, they were often not loyal to each other and stole from each other. Another participant described a risk that they perceived for these people:

*The risk for these young people who are in unstable housing situations, is that they easily get involved with the wrong people and could be drawn into drug addiction and the wrong company, and young girls are especially vulnerable and risk being drawn into prostitution for they are more vulnerable*. (Informant 3)

The professionals and volunteers also talked about how these young people often lacked phones and therefore had trouble keeping track of time and space as well as staying in touch with family and friends. The fact that they often lacked close family to engage with during holidays and be socially connected to, meant that all the volunteers and the social services were very important in giving the young homeless people a social connectedness in society.

#### 3.1.4 To suffer from mental ill-health

It emerged from the interviews with professionals and volunteers that the young people with an unstable housing situation were often suffering from mental ill-health in various ways. They tried to pretend to be strong among other people, but when meeting the professionals, they opened up about their perceived loneliness and their sadness. They were also stressed over not having a stable housing situation and sometimes suffered from blaming themselves for being in such a situation. Some were refugees, and having had their application for asylum rejected could cause stress and depression. One participant expressed it like this:

… *all the unaccompanied refugee youths should have a trauma after receiving a rejection of their application for asylum, since it's a matter of life or death*. (Informant 8)

The professionals and volunteers also highlighted the fact that many of the young people in unstable housing situations had different mental diagnoses, such as schizophrenia or psychosis, and that they often suffered from post-traumatic stress syndrome, or had diagnoses such as ADHD or autism. Some of the young people self-medicated, since they were not able to get a diagnosis due to not being sober or free from other addictions. One participant from the Swedish church described her encounters with these people:

*Young people are writing to us about their thoughts on suicide and how they are planning for suicide, but they ask: Do you have a few minutes to meet me in order to listen to me?* (Informant 12)

She also said that these young people in unstable housing situations had hit rock bottom in life and got desperate when they felt that they did not receive any more support from the society, and that they were disappointed with the social services and depressed because of their overall suffering in life.

#### 3.1.5 To struggle with healthcare and dental care access

The professionals and volunteers talked about the struggles that accessing health and dental care could entail for the young people with unstable housing situations. They were often not aware of which healthcare center they were signed into, and the professionals sometimes had to support them in calling and finding out. They often sought care within the voluntary service that the city offers, because then they did not need to pay and because they could come even if they were not sober or if they were affected by drugs. There they could also receive medication for free, for example, antibiotics, and they could be referred to the emergency care unit if needed. One participant described the encounters that these young people had had with healthcare centers:

*When these young people from the street enter the healthcare centers, they often come with dirty clothes and have not booked an appointment and get quite bad encounters with the healthcare staff, probably related to them coming in dirty from the streets*. (Informant 6)

The professionals and volunteers also said that these young people were sometimes afraid to seek healthcare, because they knew that they would be confronted about their way of living and then they probably felt like they needed to do some adjustments for their health. The interviewees also talked about how some of the young people in question were sometimes afraid of entering the healthcare center due to being scared of being reported to the police for being illegally in the country. Furthermore, the participants recounted that usually these young people waited long before seeking care, sometimes too long; if they, for example, had an injury due to an accident and waited for too long, it could be too late for any treatment.

## 4 Discussion

The results showed that the health of young people with an unstable housing situation is affected in different ways. There is a lack of basic necessities, such as food and clothes and often also a place to sleep, and the unstable situation, with its stress and lack of proper care, makes the young homeless people somatically weak. There also seem to be psychosocial challenges, due to loneliness and a lack of close contacts with family and friends, and there are problems with mental ill-health, manifesting as stress symptoms, anxiety, or mental diagnoses. Furthermore, it seems that there are challenges with regard to accessing healthcare and that these young people often hesitate to seek health or dental care, or avoid it altogether. This is due partly to the fact that some of them are in Sweden illegally and thus lack full access to healthcare, and partly to poor treatment from the healthcare system because of the homeless young people's vulnerable life situation. For this reason, some non-profit organizations offer emergency medical and dental care (20).

These results are in line with earlier research showing that homelessness is associated with worse health outcomes, both physically and mentally (21). This could, according to previous research, be particularly severe and challenging since this group of people have difficulties accessing healthcare (22). Dental health, in particular, is negatively affected by homelessness due to a lack of the necessary routines for accessing dental care. Many people experiencing homelessness also abuse drugs, which negatively affects dental health (23).

Homelessness is not merely about lacking shelter (7). Jackson and Usher maintain that it can also lead to a lack of “a vital component of [the homeless person's] sense of self” and to “stigma, marginalization and isolation,” resulting in “a state of transience and impermanence,” and a deprivation of “connection, security and belonging” (7). The stigma and marginalization were also seen in the current study, where the professionals described how young people in unstable housing situations were affected mentally due to the blame they met with—and also directed at themselves—and to sadness over the situations that they were facing. The interviewees talked about how these young people lacked close connections and contacts, which increased loneliness. Moreover, young people experiencing homelessness find it particularly difficult to get a good education, as a lack of continuity has a negative impact on schooling. Shelter accommodation makes it difficult for the young people to do their homework, due to overcrowding and lack of internet access (24).

These results are alarming in several ways. It is a human right to have a stable home, and, as mentioned above, the EU states that each country should promote access to decent housing, and prevent and reduce homelessness, for example, by making apartments accessible at lower costs (7). Even if the state should cover these aspects, it seems like the young people with unstable housing situations have to depend a lot on civic society, something which also a recent Swedish report states (25). Remedying the unstable housing situation for, among others, young people, should not be dependent on civic society; instead, society should take responsibility. However, civic society seems to cover certain needs by, for example, supplying food and clothes but also, to some extent, temporary housing, in offering shelters for homeless people. They also provide medical care and social support, which is particularly important as some of the homeless youth try to avoid health centers for fear of being stigmatized, or caught by the police.

Several of the professionals and volunteers interviewed in this study mentioned that the majority of the young people they met in unstable housing situations were migrants. We know from before that migrants already before and during the COVID-19 pandemic faced several challenges health-wise and socially, something which is unlikely to have improved. These were challenges such as lack of social contacts and lack of, or missing, family members, and challenges with regard to finding employment as well as stable housing (8, 15, 26, 27). Research during the pandemic revealed that civic society had to step in and help meet the basic needs, as well as the healthcare needs, of this group (14). Access to healthcare is often a challenge for this group in society (14, 28), as are, for instance, things like costs, not being able to understand or speak Swedish, or being excluded from healthcare if you do not have a residence permit. However, according to Swedish law, all migrants—regardless of refugee status or permit to stay—should be able to get care that is urgent (29). Healthcare staff need to be informed about this law and about the fact that they are obligated to give healthcare that cannot be deferred. If this information reached the healthcare staff and it was practiced within the healthcare centers, then probably the need for civic society to take care of migrants without a residence permit would decrease.

Recent research related to newly arrived migrants in Sweden, demonstrated how post-migration living conditions affected their mental health, highlighting both the asylum process and housing as connected to stress (30). The findings of the research recommend “context-sensitive interventions addressing individual, community, and structural factors, with a focus on improving housing conditions, alleviating day-to-day challenges, and strengthening social support networks to prevent long-term mental health issues” (30).

There is a need to target and meet the challenges related to unstable housing in a holistic way. It is about trying to solve the immediate problem of having a physical place to stay, but it is also about meeting the health needs and the social needs that accompany the unstable housing situation. This is of importance to consider in actions aimed at prevention, not least since earlier research shows an increased risk for unstable and adverse housing for young people with different mental health issues (10, 12). Another study underscored the connection between mental ill-health and social exclusion, and the significance of integrated mental healthcare for young people suffering from social exclusion, often in the shape of unstable housing situations (31). Even if the physical housing problem is solved, the people whose housing situation has been unstable will need continuous support from both the social services and civic society in order to catch up and recover from the harm that the unstable housing situation has inflicted on them. It is thus important for healthcare and social services to consider the physical, mental, and social needs that young people in unstable housing situations could have and try to meet these needs in a proper and suitable way. Healthcare services in Sweden also need to be reminded of the obligation to give all young people, irrespective of residence status, the healthcare that is urgent (29). Moreover, further research is required in relation to the perspectives of young people in unstable housing situations and how they experience and consider their living situations in Sweden.

### 4.1 Strengths and limitations

The present study has several limitations as well as strengths. One strength is that there is a cross-disciplinary research team behind the research, which increases the breadth of the research, making it possible to look at and illuminate different aspects of unstable housing situations among young people. Quite a lot of data was collected, since 19 different health, social, and civic society professionals and volunteers were interviewed and all interviews lasted between 45 and 60 min. The amount of data was therefore considered sufficient for the current study. Another strength is that the members of the research team have a great deal of experience of conducting interviews, as well as meeting the kind of professionals that were interviewed in the current study. It could, however, be considered a limitation that a wide range of different professionals were interviewed, as this could, possibly, have resulted in too wide and disparate a range of data. The chosen method for the current study was qualitative with inductive thematic content analysis, which is the most suitable method when little or nothing is known about the phenomenon under study. It is also the most common approach used to analyze qualitative data (17), and therefore no alternative method was considered.

The combined purposive and convenience sampling of the study informants, could be seen as both a limitation and a strength. In qualitative research, convenience sampling relies on the motivation of the research participants, which risks entailing motivation bias. The motivation for taking part in the research could, as formulated by Stratton, be related to “the interest one has in the research topic, a wish to express a disgruntled point of view, or desire to support one's specific opinions” (21, p. 1). On the other hand, purposive sampling could be a strength, since it could provide participants who possess rich information about the issue to be explored and who can therefore contribute important insights relevant to the purpose of the current study (18).

We have tried to make the analysis transparent by showing an extract from the analysis in an analysis schedule demonstrating how we proceeded from meaning unit to code to sub-category, which strengthens the study. All the researchers read the analysis and gave comments, which could be called inter-rater reliability (17). This led to a revision of the results, which could also be seen as a strength. Dependability was ensured by the process of data analysis and by allowing the steps of the chosen method to be followed closely (32). Credibility was ensured by the fact that an open dialogue between the authors took place during the research process in regard to decisions about the focus of the study, the selection of context, the participants, and the approach to gathering data (32). Finally, regarding transferability, the results (32) from this study could be transferable to similar urban settings, especially since many developed countries have a mixed population with several nationalities, just like Sweden, and are seeing similar challenges with regard to unstable housing for its population. However, it is always up to the reader to determine how the results from a study could be transferred to another setting (32).

## 5 Conclusions

According to the professionals and the volunteers interviewed for this study, an unstable housing situation affects young people in different ways. It affects them physically and often prevents them from being able to fulfill basic needs, such as the need of clothes and food, and of a place to sleep. It inevitably also affects their life situation as a whole as well as their future, as it makes it difficult to manage both studies and work. Some of them are also affected psychosocially, lacking close contacts and connections. Moreover, the mental health of some of the young people with unstable housing situations is impacted. In addition to this, healthcare access is a struggle for them. In order to combat all the problems that accompany unstable housing situations, a holistic approach will be necessary, along with support for those who find themselves in these situations, in order to stabilize them for the future.

## Data Availability

The raw data supporting the conclusions of this article will be made available by the authors, without undue reservation.
